# Drug Delivery Systems in Regenerative Medicine: An Updated Review

**DOI:** 10.3390/pharmaceutics15020695

**Published:** 2023-02-18

**Authors:** Alaa Mansour, Maya Romani, Anirudh Balakrishna Acharya, Betul Rahman, Elise Verron, Zahi Badran

**Affiliations:** 1 Periodontology Unit, College of Dental Medicine, University of Sharjah, Sharjah 27272, United Arab Emirates; 2 Department of Family Medicine, Faculty of Medicine, American University of Beirut, Beirut 1107, Lebanon; 3 CNRS, CEISAM, UMR 6230, Nantes Université, F-44000 Nantes, France

**Keywords:** regeneration, drug delivery, nanoparticles

## Abstract

Modern drug discovery methods led to evolving new agents with significant therapeutic potential. However, their properties, such as solubility and administration-related challenges, may hinder their benefits. Moreover, advances in biotechnology resulted in the development of a new generation of molecules with a short half-life that necessitates frequent administration. In this context, controlled release systems are required to enhance treatment efficacy and improve patient compliance. Innovative drug delivery systems are promising tools that protect therapeutic proteins and peptides against proteolytic degradation where controlled delivery is achievable. The present review provides an overview of different approaches used for drug delivery.

## 1. Introduction

Regenerative medicine is a branch of science that replaces damaged or lost body tissues or organs due to disease, trauma, or congenital defects [1]. It provides the potential to deal with healing challenges from a wide array of diseases or conditions previously considered incurable. Thus, overcoming the drawbacks associated with transplantation therapy, such as limited donor tissue supply and possibly the induction of harmful host immune reactions [2]. For this purpose, regenerative medicine applies numerous strategies utilizing cell therapy and tissue engineering and the delivery of therapeutic agents, such as drugs, proteins, and even genes, to the target tissue that contribute to the repair and healing process [3].

The fundamental elements of tissue regeneration, consisting of the extracellular matrix (ECM), cells, and a wide range of signaling molecules, are utilized by regenerative medicine either singly or in combination [4]. When the regenerative potential is impaired due to aging or systemic health problems, injecting the regenerative factor directly into the site is recommended. However, this approach is usually ineffective due to easy diffusion from the site or rapid deactivation [5]. Novel drug discoveries and advancements in biotechnology have produced macromolecules whose solubility properties and short half-life usually necessitate repeated administration [6]. Therefore, an appropriate delivery system is necessary to protect the therapeutic agent against degradation where controlled delivery is achievable.

The modification of a drug encompasses alterations in its chemical structure (e.g., functional groups and amino acid sequences) and its binding capacity to the targeting ligands. This, in turn, will modulate the interactions between the molecules and their target site and, eventually, its intended function. This process led to the discovery of new generations of remedial agents that have provided advanced therapeutic functions for the treatment of systemic diseases (e.g., cancer and invasive fungal infections) [7]. However, this increases the demand for the delivery technologies to evolve to address better the additional challenges associated with the delivery of these agents, such as low solubility, sufficient and sustained delivery, and providing efficient routes of administration.

**Figure 1:**
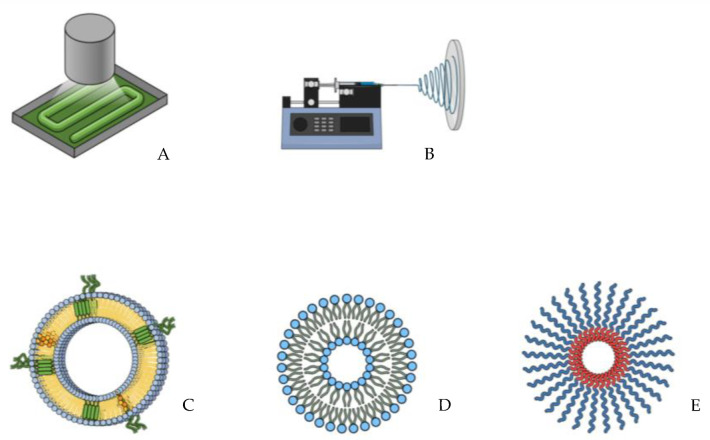
Examples of techniques and drug delivery systems tested in regenerative medicine: (**A**) 3D Bioprinting, (**B**) Electrospinning, (**C**) Extracellular vesicles, (**D**) Liposome, (**E**) Polymeric micelles.

Drug delivery scaffolds are promising tools due to their ability to maximize the therapeutic effects of the delivered drugs and bioactive agents while safely achieving their desired therapeutic effects. Those vehicles function not only to help targeted delivery to tissues or organs but also comprehensively regulate the drug’s time and dose distribution in the body to enhance the activity of the delivered agent and minimize possible associated side effects [8]. The delivery method should be typically concerned with the drug’s pharmacokinetics (including distribution, metabolism, and pharmacodynamics. With the huge diversity of the physicochemical characteristics of the pharmaceutical agents, a better understanding of material sciences and manufacturing technologies is required to ensure obtaining the appropriate dosages. The focus on noninvasive routes of administration (i.e., oral, transdermal, inhalation, and mucosal delivery) has played a key role in driving innovation in drug delivery strategies, which led to a better understanding of the drug kinetics and the biological barriers that prevent systemic drug access [9,10]. In this context, there has been a significant advancement in delivery technologies leading to innovative approaches such as nanofibers, nanogels, and micelles (Figure 1).

Furthermore, an in-depth understanding of cell biofunctions (i.e., cell-cell and cell-tissue interactions) and various types of endogenous cells have been used as delivery vehicles for treating diseases, including cancer, autoimmune, and infectious diseases. This led to setting cell-based delivery systems as a revolutionary treatment modality and presenting a fundamental change in the drug delivery field [11]. The current review was conducted to present an overview of the delivery systems used in the field of regenerative medicine.

## 2. Techniques for Fabricating Drug Delivery Vehicles

### 2.1. Three-Dimensional Printing

Three-dimensional bioprinting is an emerging strategy that facilitates the development of new drug delivery systems, implants, and scaffolds with high complexity for biomedical and pharmaceutical applications. The potential of 3D printing technology in drug delivery was first introduced in 2015 by the fabrication of the first 3D printed FDA-approved drug product known as Spritam (Levatirecam) [12]. Indeed, the 3D printing technique is based on the principle of adding successive layers to produce a digitally predesigned model. In this regard, this technology allows the fabrication of pharmaceutical products (i.e., tablets or capsules) containing a combination of drug elements and achieving varying release patterns. In this context, various 3D printed vehicles can be produced to deliver active pharmaceutical ingredients through different routes [13]. For example, 3D printed technology could be used to fabricate chewable tablets for pediatric applications that provide flexible dosing and ease of administration [14,15]. Gastric drug release can be further improved by manufacturing 3D printed tablets with retentive shells of variable thickness that show outstanding floating behavior [16,17]. For pancreatic cancer treatment, a patch fabricated using PCL could be obtained through a pressure-assisted microsyringe (PAM) printing technology to help with the local delivery of an anticancer drug [18]. Transdermal drug delivery is also assisted by 3D printing. In this regard, a customized size mask is fabricated to topically deliver drugs for treating acne [19] and as a patient-tailored antimicrobial wound dressing for the nose and ear [20]. Three-dimensional printing technology also allows the development of medical devices to help treat asthma and breathing problems [13]. Intrauterine drug delivery is also reported with the aid of T-shaped devices or vaginal rings [21].

Three-dimensional bioprinting is a rapidly growing technology that has been widely used in tissue engineering by providing the capacity to bioprinting cells, bioactive molecules, and biomaterials in a controlled layer-by-layer approach. This allows engineering tissue blocks in a 3D microenvironment with an excellent arrangement of cells to mimic their natural counterparts in structure and function [22]. The advancement in imaging technology helps create precise models for 3D printing to ensure a perfect fit into the desired tissue. It permits the replication of body organs or tissues (e.g., muscular, vascular, and skeletal systems) by depositing multiple biomaterials, cells, and bioactive molecules to create biomimetic 3D constructs Thus, offering a potential solution to the field of organ replacement and transplantation in the future [23]. Several trials of 3D tissue bioprinting have been presented, which include obtaining biomimetic structures of bone, cartilage [24], skin [25], and heart [26]. The emergence of 4D bioprinting has created novel opportunities to efficiently mimic the dynamics of native tissue and allow the delivery of drugs and cells [27]. Moreover, this technology can be used to print therapeutic proteins, making it possible to use printers in biologics [28]. The 3D printing systems include laser-based writing [29], printing-based inkjet [30], and nozzle-based [31].

Stereolithography (SLA) was the first laser-based system in which a laser beam was used to solidify a layer of a photosensitive liquid resin, and the process was repeated to build the 3D structure [32]. This technology is the most widely applied 3D printing method owing to its high resolution and reduced overall cost. It also offers the advantage of minimizing heating during the printing process, thus making it an appropriate approach for printing thermolabile pharmaceutical agents [33]. Nevertheless, an SLA-designed delivery system is usually associated with the entrapment of the active ingredient within the polymeric matrix leading to incomplete release and reduced therapeutic effect [34]. A recent advancement to the laser-based system is selective laser sintering (SLS) which precludes the use of photoreactive substances [35]. In this technique, the powder is evenly spread over the building platform, and then the laser beam is used to draw a predesigned pattern representing the finished product. The powder is finally sintered by softening a thermoplastic polymer [36]. A major advantage of this technology is the single-step printing process accompanied by high resolution without using an organic solvent [37]. However, it still retains the possibility of pharmaceutical agent degradation as a result of laser sintering [38].

Inkjet printing depends on jetting a liquid phase containing the medicine and other additives on a predetermined area to make layers until the design is built [39]. This printing system is classified based on drop generation and deposition into two major types: continuous and drop-on-demand (DOD) inkjet printing. Continuous inkjet printing involves the continuous flow of ink through an orifice to generate droplets which are then deposited onto a substrate under the control of piezoelectric transducers at the orifice [40]. Whereas in DOD inkjet printing, the formation of the droplet depends mainly on demand, and therefore it requires less ink in comparison to continuous inkjet printing [41]. The DOD inkjet printing could be further subcategorized into drop-on-liquid and drop-on-solid printing techniques. The former involves the deposition of liquid droplets under a thermal stream which results in developing microstructures with high drug loading capacity as well as appropriate for customized drug delivery. For drop-on-solid printing, the liquid droplet acts as a binder of powder particles onto which it is deposited, forming a solid structure [42]. This approach produces controlled drug delivery systems for a wide range of pharmaceutical ingredients, which can be either incorporated in the powder bed or the binder ink [43]. Nevertheless, inkjet printing presents some drawbacks, such as the low mechanical properties of the printed products due to the increased porosity and the possibility of nozzle blockage by the ink. With the high cost and limited application in printing thin film structures, further improvements are required to expand the reliability of this technology [44].

The nozzle-based system works by extruding substances from a narrow nozzle to obtain the predesigned 3D model [13]. In this technique, the solid material is mixed with the binder, and then the mixture is extruded through the nozzle to create a 3D object. Based on whether the process includes or excludes material melting, the nozzle-based system is subdivided into fused deposition modeling (FDM) and pressure-assisted microsyringes (PAM) [33]. In the FDM approach, a heated semi-liquid thermoplastic ink is extruded layer-by-layer to build a model [45]. Due to the ability to mix the heated polymer with the active pharmaceutical agent, it is possible to obtain constructs with a variety of dosages [46]. However, the PAM technique involves applying compressed air to extrude viscous and semi-liquid materials through a microsyringe which is deposited layer-by-layer into microstructures of 5–10 μm or less [33,47].

An enhancement of 3D technology can be attained by combining it with microfluidics. As a result, more sophisticated and personalized devices for drug delivery could be obtained by manufacturing 3D printed microfluidic chips. A major advantage of this technology is the possibility to fabricate particles containing poorly soluble drugs characterized by high loading capacity, sustained release, and targeted drug delivery [48].

### 2.2. Electrospinning

The electrospinning technique proved simple, versatile, and cost-effective for fabricating scaffolds with controlled release properties. Therefore, it is widely investigated for the creation of drug delivery systems [49]. Polymer nanofibers produced by this technology gained rising intention owing to their outstanding characteristics, such as flexibility, increased surface area, and the ability to facilitate cell attachment [50,51]. Electrospinning also allows producing fibers with a highly porous structure, thus increasing the surface area [52]. The small diameter of these materials permits their targeted in situ application which helps localized drug or gene delivery to the site of action and minimizes any possible adverse effects associated with its systemic perfusion [53].

Different polymers, including natural and synthetic, can be used for electrospinning [54]. Natural polymers, such as chitosan and gelatin, are similar to the physiological extracellular matrix (ECM), have exceptional biocompatibility, and have high cellular affinity [55]. However, they show poor mechanical properties and reduced stability in physiological conditions [56]. Unlike natural products, synthetic polymers, such as PCL and PLGA, satisfy the stability requirements and have excellent mechanical properties [57]. Nevertheless, their degradation results in byproducts and may be identified by the host immune system as foreign materials. Both situations may lead to the induction of undesirable inflammatory responses [58]. Alternatively, a composite of both polymer types can be produced to combine the excellent resemblance to the extracellular microenvironment with adequate mechanical properties [59].

The incorporation of drugs in the electrospun fibers is carried out through different approaches. The simplest method is the direct blending between the polymer and the embedded agent, which requires that both blended materials have similar wettability for better drug solubility and distribution in the polymer solution. The blending process is achieved by dissolving the polymer and bioactive agent in an organic solvent [60]; however, a limitation of this technique is the possible denaturing of the bioactive molecule by the solvent [60,61]. Emulsion electrospinning is an alternative approach in which droplets containing the bioactive molecule are dispersed in the polymer solution, thus enabling the formation of core-shell nanofibers by encapsulating the drug inside micelles and minimizing the contact between the bioactive molecule and the organic solvent [62]. Two factors should be considered to ensure a sustained release of the bioactive molecule, including the similarity between the polymer and the drug polarity and the solubility of the drug in the polymer solution [63].

Drug release from electrospun nanofibers can occur by either a diffusion mechanism from the nanofibers or an enzymatic reaction, resulting in drug cleavage from the system. Furthermore, drug release can be achieved by developing stimuli-responsive nanofibers, which release the bioactive agent when certain environmental conditions, such as pH and temperature [64,65]. pH-sensitive polymers are polyelectrolytes containing acidic or basic groups, which can be obtained through multiple pathways such as direct electrospinning of polyelectrolytes and composite electrospinning and surface modification of the constructs. Drug release from such constructs is regulated by the pH of the targeted tissue [66]. In comparison, temperature-responsive nanofibers undergo distinct hydrophilic/hydrophobic phase transitions at a certain temperature, which leads to their degradation and drug release [67].

Given the high loading capacity and valuable hydrophilicity of biodegradable polymers, the electrospinning of nanofibrous scaffolds has the potential for widespread applications [68]. Several drugs have been effectively delivered, including antibiotics and anticancer agents. Moreover, multiple reports demonstrated the potential for using electrospun nanofibers as nerve-guided conduits, artificial heart valves, scaffolds for bone and cartilage repair, and wound healing applications [69].

## 3. Drug Delivery Systems

### 3.1. Biodegradable Polymers

Collagen is composed of a triple helix structure consisting of polypeptide chains and gelatin [70,71]. Since it is an essential structural component of all body tissues, there has been extensive research about its potential application in regenerative medicine and, therefore, becoming the most commonly used natural polymer [72,73]. Other natural polymers, such as cellulose, chitosan, and hyaluronic acid (HA), are also being considered as potential scaffolds for tissue regeneration of different tissues, including, but not limited to, bone, cartilage, and skin [74]. On the other hand, synthetic polymers such as polycaprolactone (PCL) and poly lactic-co-glycolic acid (PLGA) are gaining popularity. They have also been evaluated for their functionality as scaffolds in regenerative medicine [75].

With the large variety of biodegradable polymers available, several factors impact their suitability to be used as a delivery vehicle. One essential criterion impacting the capacity of these polymeric materials to function as scaffolds is the content’s ease and rate of release relative to their degradation mechanisms. Optimal performance requires retaining the molecules within the delivery scaffold to be released at an appropriate rate to match the healing or growth rate of the tissue [76,77]. It was reported that this process is usually regulated through electrostatic and van der Waals forces, which can be adjusted by the pH level [78,79]. Furthermore, the polymer-lipid combination creates a delivery vehicle with increased stability and controlled release, thus, enhancing the properties of biodegradable polymers [80,81]. Other factors supporting the use of polymers in scaffold fabrication include acceptable shelf life, reasonable mechanical properties, and the production of nontoxic byproducts [82].

The properties of biodegradable polymers (i.e., biocompatibility, biosafety, and biodegradability) have been highly considered to have the potential for various regenerative applications [83]. For example, preclinical trials demonstrated that the addition of bone morphogenic protein (BMP) [84] or platelet-rich plasma (PRP) to 3D printed scaffolds ameliorates the osteogenic potential for bone regeneration [85]. A hydroxyapatite/chitosan scaffold seeded with bone marrow mesenchymal stem cells (BMSCs) supports bone regeneration by stimulating their proliferation and osteogenic differentiation [86]. Furthermore, biodegradable polymers can benefit gene delivery owing to their decreased accumulation and reduced toxic effect on targeted cells and tissues [87,88]. Nevertheless, further investigation is recommended in large animal models and clinical trials to increase the translation of those strategies and validate their use in clinical practice.

Due to the rising interest in demanding biodegradable biomaterials, natural and synthetic polymers have been investigated for a wide range of biomedical applications, including tissue engineering, regenerative medicine, and controlled drug and gene delivery [83,89]. Given its role as an initiator of the coagulation cascade combined with the thrombogenic capability, collagen could be successfully utilized as a hemostatic agent [90,91]. In addition, collagen-based scaffolds are liable to be used to deliver a variety of molecules, including drugs [92], proteins, and genes [93]. Collagen-based gentamicin-delivery vehicles (e.g., Collatamp^®^-G) are currently available and permit a sustained local delivery of antibiotics [94]. Another product (i.e., Septocoll^®^) is reported to achieve prolonged delivery by incorporating two gentamicin salts with different solubility rates [95]. In periodontics, a collagen-based chlorhexidine chip is reported to allow a more prolonged and sustained local delivery of chlorhexidine to treat periodontal pockets [96]. Furthermore, a composite of collagen and biphasic calcium phosphates (Collagraft^®^, Angiotech Pharmaceuticals) is FDA-approved as a biodegradable synthetic bone graft substitute [97]. Despite the beneficial effects of collage-based scaffolds, they show some drawbacks. One disadvantage is the immunogenic reaction to the antigenic sites in the collagen molecule, which may limit the clinical application of such materials [98]. Other limitations include the varying physicochemical and degradation properties and the risk of infectious disease transmission by collagen scaffolds obtained from xenogeneic sources [99]. To overcome these limitations, recombinant collagen peptides could be produced by different sources, such as yeasts and *Escherichia coli* [100]. However, the commercially available recombinant collagens are still expensive to produce since only limited amounts are available for medical applications [100].

Hyaluronic acid (HA) is characterized by the availability of various cell receptors and the ability to promote mesenchymal cell migration and differentiation [101]. Thus, by permitting its use in wound-dressing and tissue repair applications [102], hybrid HA scaffolds could be used for controlled and targeted delivery of anti-inflammatory and anticancer drugs [103,104]. In addition, chitosan-based scaffolds have been investigated for several applications in tissue engineering and wound healing [105,106]. Furthermore, these scaffolds have the potential for controlled and targeted drug delivery owing to their cationic nature [107]. Alternatively, synthetic polymers are gaining popularity for use as drug carriers, and these materials allow sustainable drug release and have superior mechanical properties compared to natural polymers. Moreover, it is easier to process synthetic polymers to obtain delivery vehicles of suitable pore sizes and scaffold geometries compared with natural ones [108]. Synthetic polymers could be fabricated under controlled conditions to produce scaffolds with reproducible physicomechanical properties such as tensile strength and degradation rate. Pure synthetic polymers offer reduced risks of toxicity, immunogenicity, and infections [109]. Various types of these polymeric materials, such as poly-lactic acid (PLLA) and PCL, have also been used to fabricate scaffolds for the regeneration of different tissues, including bone [110], cartilage [111], skin [112], ligament [113], and vascular tissues [114]. Due to the slow degradation rate of PCL scaffolds, they are often combined with other polymers, such as PLLA, to accelerate their erosion rate [115]. The rapid degradation properties of PLGA effectively help the delivery of different bioactive molecules, including vaccines [116,117], antibiotics [118], and anti-inflammatory drugs [119]. Furthermore, polyurethanes have been used extensively and applied in developing prostheses such as cardiac assist devices [120], vascular shunts [121], and tracheal tubes [122].

### 3.2. Nanomedicine

Multiple limitations are still encountered with the traditional drug-delivery systems, with special attention to efficacy and achieving an adequate level of patient compliance. The introduction of nanotechnology has offered a novel strategy for drug delivery as it resulted in the development of novel carriers capable of releasing not only a wider range of molecules but also allowing for a more specific targeted delivery with improved controlled release properties. Examples of these nanocarriers include extracellular vesicles, liposomes, inorganic particles, and polymeric micelles [123].

### 3.3. Extracellular Vesicles

An early study illustrated that extracellular vesicles (EVs) are nanoscale lipid-bound structures secreted into the extracellular space, which include three main subtypes named exosomes, microvesicles, and apoptotic bodies [124]. Exosomal vesicles are 30–150 nm in diameter and represent a homogenous population of endocytic origin formed by inward budding of the multivesicular body (MVB) membrane. Microvesicles typically range from 100 nm to 1 µm in diameter. Their route of biogenesis is thought to necessitate certain cytoskeleton components such as actin and kinesins. In comparison, apoptotic bodies are a group of nanostructures released by dying cells into the extracellular space [125]. The main characteristic of these structures is the inability to replicate as they do not contain a functional nucleus [124]. However, they have a complex content consisting of proteins, nucleic acid species (i.e., mRNA and DNA), and lipids [126]. They are found in different cells, such as lymphocytes, platelets, macrophages, epithelial cells, fibroblasts, and stem and cancer cells. EVs have also been detected in various biofluids such as milk, blood, plasma, amniotic fluid, and saliva [127]. Given their biocompatibility, inherited ability to cross major biological barriers (e.g., blood-brain barrier), and capability to transmit biological signals between cells [128], EVs have been considered in the context of regenerative medicine and delivery systems [129,130].

An early study demonstrated the presence of a variety of minute products that could be obtained from the breakdown of RBCs [131]. There is rising evidence that EVs play a key role in regulating normal physiological processes such as tissue repair and blood coagulation [132]. Indeed, these structures possess surface reports that facilitate binding with the plasma membrane of the recipient cells, followed by delivering their effector molecules (e.g., RNA). EVs can stimulate the pathological mechanism involved in the incidence and progression of several diseases, such as diabetes mellitus, obesity, and tumor progression [133,134]. Chemotherapeutic drug delivery to the target site is challenging, and it is usually accompanied by serious side effects. EVs offer an effective approach to overcoming such limitations. Indeed, studies demonstrated the capacity of EVs to provide high drug concentration at the target site, which improves the therapeutic effect while reducing systemic toxicity. For instance, EVs loaded with DOX or PTX exhibit improved targeting and anticancer effects. Furthermore, EVs have gained much attention as nucleic acid delivery carriers as they overcome the limitations associated with their use, such as immunogenicity and the inability to penetrate physical barriers [135].

Two main factors have been shown to influence the therapeutic outcomes obtained with EVs. The origin of EVs seems to play a crucial role in maximizing the benefits and minimizing any potentially harmful responses [136]. For example, the use of autologous EVs may be more favorable for therapeutic purposes due to the possibility of inducing immune reactions with the heterologous (i.e., allogenic) ones [137]. It was also shown that tumor-derived EVs carry antigens specific to the originating tumor cells; therefore, by delivering these agents to immune cells (e.g., dendritic cells), an immune reaction can be developed against cancer cells [138,139]. Immune cells such as macrophages represent another important source for EVs owing to their ability to evade phagocytosis, which is a major limitation for most of the other types of EVs, allowing for longer persistence in circulation and improved efficacy [140,141]. Preclinical studies revealed the effectiveness of those derived from bovine milk against lung cancer; however, they lack specificity to the recipient cells [142]. The other factor is the method of administration of EVs. The rapid clearance following the localized or systemic injection necessitates the development of approaches to improve the EVs’ retention at the target site and avoid multiple therapeutic doses application. In this regard, biomaterial-based delivery scaffolds fabricated using sponges or hydrogels have been used to help prolonged release and improve therapeutic efficacy [143].

EVs can be isolated from different sources via various techniques, including differential ultracentrifugation, chromatography, density gradient centrifugation, and microfluidic device [144]. Therapeutic agents (e.g., proteins, nucleic acids, and drugs) can be loaded into EVs through two approaches. One is “in vitro loading”, in which the therapeutic cargo is first incorporated into donor cells before the EVs’ isolation [145]. The other approach, ex-vivo loading, involves loading after isolation via various methods, including electroporation, incubation, extrusion, or permeabilization [146].

The therapeutic potential of EVs has been reported in multiple studies. For example, these nanostructures showed the capability to stimulate cell proliferation, induce angiogenesis [147], and act as a carrier of immunomodulatory signals [148]. In the field of regenerative medicine, EVs have been investigated for their beneficial role in regulating tissue regeneration. In this regard, in-vivo models demonstrated the therapeutic capacity of mesenchymal stem cells (MSCs)-derived EVs in protecting against kidney damage [149], repairing skin defects and wounds [150], and stimulating triggering cardiac repair following myocardial infarction [151], and the improvement in cardiomyocyte and endothelial cell survival and decreasing the oxidative stress pathway [152]. Another animal model reported the therapeutic role of EVs in regenerative medicine, especially in reducing LPS-induced acute respiratory distress syndrome (ARDS), which suggests the potential of this modality in controlling the inflammatory response and fibrotic consequences following COVID-19 infection [153]. Other potentials of EVs have been demonstrated in models with kidney [154], neurological [155], and hepatic diseases [156].

### 3.4. Liposomes

The first reports were published in 1965, describing the detection of swollen phospholipid structures, which were then identified as liposomes [157]. These microscopic structures consist of concentric lipid bilayers enclosing aqueous spaces [158]. Due to their structural features, liposomes are characterized by outstanding properties that provide enhanced protection, controlled release, and extended half-life of encapsulated substances [159]. Furthermore, they have a unique capability of carrying both hydrophilic and hydrophobic molecules. The bilayer phospholipid membrane possesses a hydrophobic tail and a hydrophilic head, resulting in the amphipathic nature of liposomes. This structure allows the entrapment of both hydrophilic (polar) and hydrophobic (nonpolar) compounds. As drug vehicles, liposomes present some outstanding properties making them drug transport options, such as the enhanced ability to protect the encapsulated substances from degradation and extend their half-life, and control the release of drug molecules at the target site. Furthermore, liposomes can efficiently deliver the loaded substances at the disease site through a passive or active release that could help decrease the systemic side-effect and improve therapeutic benefits [160]. Therefore, liposomes are the highly investigated nanocarriers for delivering a wide range of drugs and other small molecules such as protein, nucleic acid, and imaging agents [161].

Nevertheless, the sterilization process can easily impair the stability of these structures due to sensitivity to high temperatures and certain types of radiation [159,162]. Liposomes are also prone to rapid clearance from the circulation by phagocytes, thus, affecting the duration of the agent delivery [160]. Different classifications of liposomes have been reported. Based on lamellarity, liposomes are either unilamellar vesicles (ULV) or multilamellar vesicles (MLV) [159]. Regarding composition, liposomes are available as conventional, cationic, long-circulating, and immuno-liposome [160].

Liposomes can be prepared through various approaches, including thin-film hydration, solvent injection, and double emulsion technique [159]. The film-hydration method produces a thin film by evaporating the lipid–solvent solution under a vacuum [159]. In comparison, the solvent injection method involves dissolving lipophilic substances in an organic solvent, followed by the injection into an aqueous buffer to form liposomes [163]. On the other hand, the double emulsion approach includes the formation of the water-in-oil emulsion and then solvent extraction and microfiltration to ensure the removal of the free drug [164]. The bioactive molecule (e.g., drug and proteins) could be loaded either during the preparation of the liposome (i.e., passive approach) or after the completion of the preparation process (i.e., active method) [159].

The delivery of loaded molecules can be achieved via four types of liposomal delivery systems, including conventional liposomes, ligand-targeted liposomes, sterically-stabilized liposomes, and a combination [165]. Conventional liposomes were the first to be used, providing enhanced distribution of the delivered agent; however, this delivery vehicle can be rapidly eliminated from circulation leading to its limited therapeutic efficacy [165]. To compensate for this drawback, sterically-stabilized liposomes were introduced with improved stability properties [166]. The potential for site-specific delivery could be provided through ligand-targeted liposomes [167].

Due to the unique structure of liposomes, research interest has been given to benefit from their characteristics to discover new applications. These nanostructures offer the advantage of stability and could be combined with scaffolds to overcome the issues of toxicity. In this context, liposome-containing scaffolds may be used to deliver genes in an efficient, cell-controlled, and localized manner for multiple tissue engineering applications [168]. For instance, the delivery of genes encoding osteogenic growth factors, such as TGF-B and BMP, is becoming one of the clinically relevant applications for bone regeneration. These strategies can help initiate progenitor cell differentiation and regulate the pathways related to stimulating new bone formation and maturation [169]. It has been reported that the microtubule-stabilizing agent paclitaxel (PTX) can reduce scarring and improve axon regeneration following spinal cord injury. To make benefit from this, a collagen scaffold of PTX-loaded liposomes is designed to permit the prolonged release of PTX to stimulate neural stem cell differentiation and sensory neuron regeneration [170].

### 3.5. Inorganic Nanoparticles (NPs)

In addition to the lipid-based structure, NP can be synthesized using inorganic materials (e.g., silver, gold, and iron) and engineered into different sizes and geometries for various drug delivery applications [171]. Furthermore, these structures’ unique magnetic and radioactive characteristics permit their use in diagnostic and imaging applications [172]. They also possess several advantages, such as good biocompatibility and versatility for surface functionalization [173]. However, the low solubility and the possible toxic effect put some limitations on the clinical applications of inorganic NP [174].

Sliver NP shows a wide range of biomedical applications owing to their valuable properties. For example, they are involved in the composition of antiseptic products due to their antimicrobial influence [175,176]. Their ability to affect the cell cycle and apoptotic pathways could be beneficial in managing cancers such as leukemia [177,178]. However, the rapid elimination of sliver NP by the reticuloendothelial system, specifically macrophages, can limit their use in this field [179,180]. Gold NPs are approved by the FDA for use in various biomedical applications owing to their low toxicity, biocompatibility, easy surface modification, and controlled drug release [181]. Drugs can be conjugated to gold NP through simple physical absorption or via ionic or covalent bonding and then delivered to the target area in response to biological stimuli or light activation [182]. Iron oxide is another used for synthesizing magnetic inorganic NPs composed of magnetite (Fe_3_O_4_) or maghemite (Fe_2_O_3_). These structures possess superparamagnetic properties, thus, permitting their application as contrast agents for imaging tumors [183]. They can be further applied for drug delivery and thermal-based therapeutics [184]. Other common inorganic NPs include calcium phosphate and silica NPs, and both have shown promising outcomes when used for gene and drug delivery [185].

Zinc oxide (ZnO) has gained much research interest due to its chemical and mechanical stability rendering it an appropriate metal for pharmaceutical applications. Accordingly, ZnO nanoparticles have been investigated as a drug delivery vehicle and have been approved by the FDA, given their biocompatibility and nontoxic nature. These nanostructures exhibit antibacterial and anticancer activity linked to their capability to generate reactive oxygen species (ROS) [186]. Drug-loaded ZnO NPs can penetrate the cells of the targeted tissue through various endocytic pathways; therefore, they have emerged as an effective nanocarrier for drug delivery. The release of the cargo is regulated by various stimuli such as temperature, light, and pH [187,188]. Drug-loaded ZnO NPs have shown a significant impact in treating cancer involving different organs such as the lung, liver, breast, and blood cells (i.e., leukemia) [189]. It seems that ZnO NPs could be conjugated into antidiabetic agents for the treatment of insulin and non-insulin-dependent diabetes. Other beneficial applications have been demonstrated for the treatment of fungal diseases and various forms of bacterial infections [188,190].

Selenium (Se) NPs are characterized by low toxicity and high degradability, and immunomodulatory capability by targeting macrophages and regulating their polarization [191]. In drug delivery, Se NPs can be conjugated with high concentrations of various types of agents, which enhances their concentration at the targeted site of delivery. For example, the drug delivery capacity of these nanoparticles shows an effective application in cancer therapy and enhanced killing efficiency against bacteria [192,193]. Magnesium oxide (MgO) NPs have also attracted interest as therapeutic and potential drug carriers because of their biodegradability, biocompatibility, and nontoxicity for human cells at concentrations under 300 μg/mL [194]. In this regard, MgO NPs show effective application for a controlled system of drug delivery for anticancer drugs such as albumin and doxorubicin [195]. Copper oxide (CuO) nanoparticles possess various properties and have diverse applications, including biomedical (e.g., antimicrobial, antifungal, antioxidants, anticancer, and drug delivery), thermosensing, gas sensors, and high-temperature superconductors [196]. Copper oxide nanoparticles could be obtained through chemical or physical approaches; however, they demonstrate some limitations, such as the use of highly toxic chemicals that may cause unfavorable effects in medical applications, high cost, and high energy consumption. Alternative green routes can be used for nanoparticles synthesis (e.g., plant, fungi, and bacteria) that are eco-friendly and cost-effective [197].

The development of nanoparticles-loaded biomaterials introduces a new class of biomaterials with improved biological performance (e.g., enhanced cell adhesion and differentiation) and mechanical properties for different regenerative medicine applications [198]. Various inorganic nanoparticles could be integrated within polymeric matrixes. For instance, the incorporation of gold and bioactive glass nanoparticles within a polymeric scaffold produces an injectable composite gel for bone regeneration [199]. In addition, a biomimetic nanofibrous scaffold containing TiO2 nanoparticles shows high efficacy as a wound dressing material in tissue engineering applications with the capability to promote cell adhesion and proliferation [200].

### 3.6. Polymeric Nanoparticles

These structures can be synthesized from natural or synthetic materials giving a wide variety of possible constructs with different characteristics [171]. Various techniques could be applied to obtain polymeric nanoparticles, such as emulsification, ionic gelation, and nanoprecipitation [201]. Therapeutic agents are loaded to nanoparticles through direct encapsulation within the core, chemically conjugated to the polymer, or simply bound to the particle surface. The loading and release efficacies can be modified by modulating the properties of polymeric nanoparticles, such as composition and surface charge [202]. Different forms of polymeric nanoparticles are available such as polymersomes and micelles.

Polymersomes are artificial vesicles having membranes made using amphiphilic copolymers characterized by improved stability and cargo-retention efficiency, making them effective for therapeutic agent delivery [203]. Furthermore, polymersomes as nanoreactors possess the capacity to shield molecules such as enzymes or proteins in nanometer-size compartments while preserving their functionality in situ [203]. Polymeric micelles, which represent another form of nanostructures, are made of amphiphilic copolymers with a hydrophobic core that can be loaded with hydrophobic drugs and a hydrophilic shell that stabilizes the core and facilitates the solubility of these structures in water [204]. These nanostructures can be obtained by directly dissolving polymer material into a solvent, followed by a dialysis process or precipitation by adding a solvent [204]. Drugs can be loaded within polymeric micelles by three methods, including direct dissolution, solvent evaporation, and the dialysis process. For the direct dissolution process, both polymer and drugs are simply combined or with the aid of a water medium to form a drug-loaded micelle. While in the solvent evaporation process, the polymer and the drug are dissolved in a volatile organic solvent. However, with the dialysis process, the polymer is first dissolved in the organic solvent, combined with the drug, and then dialyzed to produce the micelle [205].

In general, polymeric NPs are ideal vehicles for drug delivery owing to their biodegradability, biocompatibility, and storage stability. Furthermore, surface modifications can be performed, which expand their functions to deliver drugs, proteins, and genetic material to targeted tissues. Nevertheless, the disadvantages of polymeric NPs include an increased risk of particle aggregation and toxicity [201]. In the field of regenerative medicine, it has been demonstrated the potential of polymeric nanoparticles as they possess improved transport properties owing to their ability for deep tissue penetration, which permits more efficient delivery of therapeutic agents to the site of action. One example of their application in tissue engineering includes the feasibility of delivering growth factors to stimulate osteoblast differentiation and bone tissue regeneration. Another approach involves the delivery of genetic materials to influence the production of certain proteins for tissue engineering [206].

### 3.7. Cell-Based Delivery System

Drug delivery systems have been utilized to introduce immunotherapeutic agents (e.g., immune checkpoint inhibitors) to the target site for initiating an immune response. However, since most current drug delivery systems are prepared with inorganic or organic materials, there is an increased potential for rapid clearance caused by immunogenic reactions and long-term toxicity to the tissues, which limits their clinical applications [11]. Endogenous cells play an important function in biological systems as carriers for proteins and molecules while demonstrating low toxicity and immunogenicity [207]. Compared to traditional delivery systems, a cell-based delivery approach offers improved biocompatibility, reduced risk of toxicity, an excellent capability of crossing biological barriers, and enhanced site-specific delivery [208]. This attracts attention toward cell-based drug delivery systems as an effective modality for the treatment of various types of cancer, such as leukemia and ovarian cancer [209], as well as antiviral therapy [210]. Examples of cell-based delivery technology include red blood cells (RBCs), neutrophils, and dendritic cells (DCs).

RBCs are the most commonly used carriers due to their abundance, relatively long half-life compared to other blood cells, wide surface area for drug assembly on the surface, and the absence of cell nuclei providing enough space for intracellular drug encapsulation [211]. Additionally, these cells possess a variety of natural surface biomarkers (e.g., CD47) that help avoid clearance by phagocytosis [212]. Despite their efficacy, some issues should be considered when using RBCs as a delivery system. Surface conjugation of drugs reduces the elasticity and deformability of RBCs, leading to accelerated clearance by the endothelial reticulum system and a decreased circulation time [213].

In response to pathogen invasion into tissues, neutrophils migrate from the circulation to the site where inflammation occurs. This chemotactic phenomenon inspired researchers to use neutrophils as natural carriers to target inflammation sites. For this purpose, drugs are usually delivered through this system by surface conjugation of the drugs or internalized by neutrophils via coculture methods [214]. DCs have a group of functions in the immune system, including antigen presentation to T cells and secreting cytokines that promote the accumulation of T cells and enhance their killing activity [215]. Given these properties, the DCs-delivery system has been developed to deliver antigens and activate the immune system for cancer treatment [216].

## 4. Discussion

With the variation in 3D printing techniques, including inkjet printing, fused deposition modeling, material extrusion, and stereolithography, a wide range of constructs with different formulations could be produced, which provide extreme benefits in biomedical applications. During the last decades, a significant amount of research has been conducted to augment the efficacy of this technology by producing 3D printed scaffolds while reducing material waste [217]. Obtaining more regulated formulations with high dose precision becomes feasible owing to the high resolution of the printing techniques, which also permit fabricating low-coat medication devices that help patients with chronic disease get therapy at an affordable price. Accordingly, 3D printing plays a crucial role in achieving significant advancement in the biomedical field and health care. The application of 4D printing is a novel strategy that offers a substantial advancement in the pharmaceutical sector by offering printed scaffolds with improved controlled drug delivery properties. This could be achieved through the fabrication of smart devices affected by stimuli such as pH, which in turn, provides superior drug release and absorption at the site of action, thus improving treatment efficacy [218].

Injectable polymers such as polyesters, glycolic acid, and caprolactone are biodegradable and biocompatible materials that are usually the preferred choice for localized drug delivery and regenerative medicine due to their tunable and sustained release properties and the mitigated surgical needs for their application. These polymers are considered safe as they degrade into natural fatty acids and hydroxy alkanoic acids [89]. Therefore, these injectable polymers have been recommended as drug carriers, tissue fillers, or scaffolds for regenerative medicine. For instance, polymers have been used as fillers that may remain in tissue for a year or longer and provide wrinkle-free skin. In contrast, others could be used for extended delivery of anticancer agents to the affected organ or antibiotics to the bone to eradicate acute or chronic bone bacterial infection. In fact, injectable pastes allow the extended-release of VEGF for tissue regeneration [219].

Polymer-based fibers can be fabricated using the electrospinning technique, and the experimental conditions can be optimized to design fibrous constructs with variable fiber size and alignment essential for drug delivery [220]. It has been shown that electrospun constructs are characterized by their high drug-loading capacity and tunable release properties, which can be further controlled using various external stimuli such as light and temperature [221]. Therefore, fabricating electrospun fibers exhibiting controlled release properties seems to have the efficacy to maintain the therapeutic dose at the site of action. In this regard, a wide spectrum of drugs, such as antimicrobial, anti-inflammatory, and anticancer, can be loaded using these scaffolds without affecting their bioactivity [54].

Nanotechnology is becoming the driving force for the evolution in the medical field, rendering nanomedicine one of the areas of research interest during the last decades [205]. In the current review, different types of nanostructures are presented, which have been demonstrated to offer a modern platform for drug delivery that greatly help improve the efficacy of available therapeutics and enable the creation of entirely new therapeutic entities. In addition, nanoparticles give a chance to deliver the predetermined amount of drug to the affected cells without disturbing the physiology of the normal cells. Cancer therapy is one of the major applications of nanoscale carriers to minimize toxicity in healthy organs by specifically targeting cancerous cells [222]. Nanoparticle-based drug delivery combined with cell therapy can also achieve a more complete and potent regenerative response [223]. Indeed, emerging biomaterials are tailored to provide a microenvironment and tunable properties that optimize the therapeutic influence and the fate of transplanted cells and improve current cell delivery strategies [224].

Cell-based delivery systems derived from circulating cells, such as RBCs and immune cells, are promising candidates for drug delivery due to their biocompatibility, non-immunogenicity, long circulation times, and barrier-crossing capability [208]. Nevertheless, some aspects still require consideration to achieve excellent drug delivery outcomes. First, to avoid causing toxicity to the carrier cells, the drug should not be freely encapsulated within the cells and alternatively loaded inside a vehicle such as liposomes or microparticles. Isolating cells, drug loading, and reinjecting cells back into the patient may alter the cell properties [225].

## 5. Future Directions

The healthcare sector is always looking for substantial development to improve the biomedical services provided as well as the patient’s health and quality of life. In this regard, the integration of 3D printing technology into clinical practice could pave the way for a health revolution through the advancement in the design of medicines and delivery of therapeutic agents to the site of action. Indeed, there is clear evidence of the continuous growth of such a delivery system, and it is becoming necessary to translate the theoretical benefits of 3D printing into real-world benefits for patients.

With the growing evidence on the efficacy of biodegradable polymers, their clinical application is yet to be investigated. They reveal valuable utilization for the localized delivery of therapeutic agents, especially into tumors and infected tissues. However, the use of these polymers should be extended to include the systemic delivery of biomolecules such as proteins and peptides. This gives the impression that biodegradable polymers will revolutionize the field of controlled drug delivery and tissue engineering in the upcoming decades.

Interestingly, it has been reported that complex biomolecules could be successfully encapsulated using the electrospinning technique, and their controlled delivery has the potential to enhance cell attachment and proliferation at the site of administration. In addition, a combination of different drugs and/or growth factors can be simultaneously loaded and delivered into the therapeutic site using a 3D fibrous construct which is advantageous to induce the rate of the damaged tissue healing. Electrospun cell patches loaded with biomolecules seem to be a promising approach in cardiac tissue engineering, and implantable drug-loaded electrospun fibers show excellent performance in vivo models. However, further trials should be conducted to validate the efficacy and success of such applications. Overall, it is becoming evident that electrospinning is a promising technology for delivering various drugs and biomolecules for functional tissue engineering.

For further improvement of nanotechnology, further investigation should be focused not only on the design of the delivery system but also on overcoming its possible drawbacks, such as systemic cytotoxic effects and immune responses of these systems. This would better elucidate the biocompatibility of nanoscale delivery systems and will direct future studies. Despite increased research efforts made to improve the outcomes obtained with the aforementioned nano-delivery systems, there are still aspects that limit their clinical application, such as the lack of standardization criteria to characterize these novel systems and the necessity of conducting preclinical studies on animal models with a specific disease to validate their use for treating such conditions. A better understanding of the molecular signature of disease will allow enhanced targeting without altering the normal cellular process. In fact, one major objective of future research should be focused on achieving this aspect which is paramount toward achieving a remarkable advancement and wider application of nanomedicine.

Finally, cell-based systems are still growing, requiring further conduction in vivo studies to evaluate the fate of cell-mediated drug delivery systems to optimize their design and unveil the appropriate administration route. With the continuous advancement, it may be anticipated that cell-based systems DDSs will demonstrate an effective means for the precise treatment of diseases.

## 6. Conclusions

The emergence of novel platform technologies for drug delivery provides significant insights into their possible applications. The quick evolution of therapeutic landscapes, delivery strategies, and technologies shows robust development to be consistent with changing drug delivery needs. These innovative and smart drug delivery systems can cross biological barriers, provide prolonged and controlled drug release at the target site, and incorporate multiple drugs within the same delivery vehicle. Their application plays a crucial role in minimizing drug toxicity, increasing bioavailability, and reducing dosage frequency, thus, helping to improve the patient’s compliance and quality of life. Accordingly, the advancements in this field will offer a valuable contribution to improving drug treatments and reducing side effects.

Biodegradable polymers have excellent characteristics, including ease of application and biocompatibility. Other factors evolve their potential and suitable use as scaffold material in regenerative medicine, such as the possibility to combine various types of signaling molecules and cells. In general, in comparison to synthetic polymers, natural biomaterials possess better biocompatibility but inferior mechanical properties. For this reason, it seems more beneficial to fabricate customized hybrid polymeric structures with improved properties for specific biomedical applications. To help do this, recent reports addressed a variety of techniques that open the door for researchers to easily synthesize and engineer scaffolds with the best specifications and properties to match the desired biomedical function and the targeted tissue.

Indeed, there is an increased interest in developing scaffolds acting as a biomatrix for active pharmaceutical ingredients with enhanced release properties. In this regard, 3D bioprinting is an evolving technology to generate such a scaffold by incorporating polymeric biomaterials as the bio-ink. This offers flexibility in the carrier design and is an on-demand and cost-effective production. This makes it feasible to produce a wide variety of pharmaceutical formulations, dosages, and customized drug delivery systems. Three-dimensional bioprinting techniques permit generating of scaffolds with improved properties such as cell distribution, fluid flow, and porosity. The advancements in polymer synthesis techniques make it feasible to develop polymer combinations to create materials that possess desired properties for highly specific applications. Current advances in 3D technology, in conjunction with increased research in this field, will help assure more safe drug delivery system and effective treatment.

Electrospinning is an exceptional technology for the development of innovative drug delivery systems able to maximize the therapeutic benefits of drugs, minimizing, at the same time, their undesired side effects. Electrospun nanofibers offer crucial benefits for different biomedical applications, given the ability to encapsulate hydrophilic and hydrophobic agents as well as their porous structure, which allows diffusion of entrapped agents. The properties of a polymeric material can be further tuned by changing the mechanical properties or the kinetic release to commensurate with the needed requirements for a specific application. Therefore, electrospun scaffolds provide a new horizon for personalized medicine.

Nondimensional materials outperform the traditional systems in regard to drug delivery in the live system, and the future advancement of drug-delivery systems necessitates further research and understanding of the application of nanotechnology. Systems based on nanostructures are used to overcome solubility issues and to enable the delivery of small molecules to their site of action while reducing off-target side effects. Liposomes are available in different sizes and shapes and possess beneficial characteristics as a drug delivery system. However, their interaction with the cells mainly influences the efficacy of drug delivery. Nanoparticles provide promising platforms for therapeutic and imaging agents with a wide variety of shapes, sizes, and surface properties that allow an optimized delivery for a specific application. Polymeric micelles have also emerged as a fascinating type of drug that carries -pH, light, and temperature-sensitive properties to facilitate programmed drug delivery. The application of nanomedicine in drug delivery will certainly remain the future field of research interest. Therefore, a continued search is recommended to develop and manufacture those nanostructures to enhance their cellular interactions and improve drug-target selectivity.

The patient’s cells present a versatile platform of carriers for the delivery of therapeutics to the target sites. Even though there is a significant role of cell-based delivery systems for immunotherapy, there are still challenges that require further advancement. First, the possible hyperactivity of the immune system would put the safety of cell-based immunotherapy at risk. More importantly, the nonspecific distribution of injected cells may induce toxic effects, especially against healthy tissues.

## Data Availability

Not applicable.
